# Patient Preference Distribution for Use of Statin Therapy

**DOI:** 10.1001/jamanetworkopen.2021.0661

**Published:** 2021-03-15

**Authors:** Suzanne Brodney, K. D. Valentine, Karen Sepucha, Floyd J. Fowler, Michael J. Barry

**Affiliations:** 1 Informed Medical Decisions Program, Massachusetts General Hospital, Boston; 2 Health Decisions Science Center, Massachusetts General Hospital, Boston; 3 Center for Survey Research, University of Massachusetts, Boston

## Abstract

**Importance:**

Thresholds for initiating statin therapy should be informed by patients’ preferences.

**Objective:**

To define the preference distribution for statin therapy across the spectrum of cardiovascular disease (CVD) risk after participants were informed about the benefits and harms of statin therapy.

**Design, Setting, and Participants:**

A cross-sectional survey was conducted from May 13 to June 2, 2020. Participants included 304 individuals aged 40 to 75 years drawn from a nonprobability opt-in panel who had not taken a statin or proprotein convertase subtilisin/kexin type 9 inhibitor in the past 3 years and knew the results of their total cholesterol, high-density lipoprotein cholesterol, and blood pressure measurements.

**Exposures:**

Personalized 10-year CVD risk with and without statin therapy and potential harms of statins.

**Main Outcomes and Measures:**

The primary outcome was self-reported preference for statin therapy.

**Results:**

The 304 participants had a mean (SD) age of 54.8 (9.9) years; 152 were women (50.0%), 130 (42.8%) non-White, 50 (16.6%) had a high school degree or less education, and 153 (50.8%) reported never needing help reading health materials. When asked their preference for using statin therapy after reviewing their benefit and risk information, 45% of the participants reported they would definitely or probably choose statin therapy. As the risk increased, the proportion who would choose statin therapy generally increased (from 31.1% for a risk <5% to 82.6% for a risk >50%). The minimum risk threshold had to increase to 20% before 75% of respondents in that risk group would want statin therapy. For participants with a risk greater than 10%, the desire to use statin therapy decreased as participants’ health literacy, subjective numeracy, and knowledge scores increased.

**Conclusions and Relevance:**

In this study, preferences for statin therapy for primary prevention of CVD appeared to vary across the spectrum of 10-year cardiovascular risk, but they were relatively flat at intermediate levels of risk. This preference distribution suggests a broad risk range for shared decision-making.

## Key Points

**Question:**

What are patients’ preferences for use of statin therapy for primary prevention across the spectrum of estimated 10-year atherosclerotic cardiovascular disease risk?

**Findings:**

Of the 304 adults who participated in this survey study, the proportion who would want statin therapy after reviewing personalized benefit and harm information was 51% or less for risk categories under 20%. The minimum risk threshold had to increase to 20% before 75% of respondents in that risk group would want statin therapy.

**Meaning:**

The results of this survey did not note a risk threshold that would justify a standard for or against the use of statin therapy; the preference distribution suggests a broad range of atherosclerotic cardiovascular disease risk to recommend shared decision-making.

## Teaser

This survey study examines the preferences of patients for use of statin therapy in prevention of cardiovascular disease based on the participants’ understanding of the benefits and risks associated with use of the medications.

## Introduction

In the early years of clinical practice guidelines, Eddy^1^ published an influential series of articles describing an approach to the development of what he called practice policies. He described 3 levels of policies with increasing flexibility: standards (must be followed), guidelines (should be followed), and options (neutral recommendation). He highlighted the need not only for outcome probabilities for management strategies but also patient preferences. He stated that a policy for or against an intervention should not be a standard unless “…at least 95%, perhaps even 99%, of people who are candidates for the intervention should agree on the desirability of its outcomes.” For a guideline, “…an appreciable but not unanimous majority of people…..might be said to exist if 60% to 95% of people agree on the overall desirability (or undesirability) of the outcomes.”^1^^(p3181)^ Although these thresholds are arbitrary and arguable, the importance of knowing patient preferences for the development of guidelines is the key point. Eddy^2^ also proposed a practical approach to eliciting these preferences from potential patients who would be the target of the guideline by providing a balance sheet detailing the outcomes of an intervention and its alternatives.

Nevertheless, the formal incorporation of patients’ preferences into guidelines was never established, in part owing to the time and expense of collecting such data, which were rarely available in the literature. Instead, guideline development focused on estimation of outcome probabilities; rating the quality of those data; and using guideline panel members, mostly clinicians, to develop recommendations using their subjective sense of patient preferences.

Guidelines for the treatment of hyperlipidemia with statins for the primary prevention of cardiovascular disease (CVD) are a good case in point. Two of the most widely used guidelines in the US are from the American College of Cardiology and the American Heart Association^3^ and the US Preventive Services Task Force.^4^ Both guidelines suggest that clinicians assess patients’ 10-year risk of a CVD event using the pooled cohort equations for adults aged 40 to 75 years. While emphasizing the need for a risk discussion between clinician and patient across the spectrum of risk, these 2 guidelines differ on when to discuss or initiate statin therapy. The American College of Cardiology and the American Heart Association guideline recommends discussing statin therapy starting at a 10-year CVD risk of greater than or equal to 7.5%. The US Preventive Services Task Force guideline recommends initiating statin therapy with 1 or more CVD risk factors and a 10-year CVD risk greater than or equal to 10%. A new guideline from the Veterans Affairs and Department of Defense introduces a strong recommendation for offering moderate-dose statin therapy with a 10-year CVD risk of greater than or equal to 12%.^5^ To our knowledge, none of these thresholds were informed by the preferences of patients for statin therapy.

The purpose of our study was to describe people’s preferences for statin therapy who were not taking a statin or proprotein convertase subtilisin/kexin type 9 inhibitor for primary prevention across the spectrum of estimated CVD risk after informing them about their personalized benefits and harms of statin therapy. We examined whether clinicians’ previous recommendations about statins or respondents’ educational level, health literacy, numeracy, or knowledge were associated with their preferences.

## Methods

From May 13 to June 2, 2020, we contracted with Qualtrics to conduct a survey drawn from a nonprobability opt-in panel with quotas applied during selection for age, sex, race, ethnicity, and educational level to achieve a diverse convenience sample of 309 respondents. The Mass General Brigham Human Research Committee Institutional Review Board approved this study as exempt as a low-risk survey study. Respondents accessed the survey from 42 different states. Participants opted in to complete the survey after reading a consent form and received compensation. Eligible people were aged 40 to 75 years who had not taken a statin or proprotein convertase subtilisin/kexin type 9 inhibitor in the past 3 years and knew the results of their total cholesterol, high-density lipoprotein cholesterol, and blood pressure measurements. Participants self-identified their race and ethnicity. Individuals were excluded if they were allergic to a statin or if they had an indication for a statin for secondary prevention: a prior CVD event, including a myocardial infarction, stroke, blockage in a blood vessel in the neck or head, or a coronary artery bypass operation or a stent placed in 1 or more arteries. The survey was constructed so questions could not be skipped. Rather, each question had a response option indicating the participant did not want to answer. Quality control checks included setting a minimum amount of time to complete the survey (6 minutes).

To calculate a personalized estimate of each respondent’s 10-year CVD risk, respondents were asked to enter their age, sex, total cholesterol and high-density lipoprotein cholesterol levels, systolic blood pressure, any treatment for hypertension, whether they had diabetes, and smoking status into an online risk calculator that used the pooled cohort equations.^6^ Since the risk calculator was publicly available, respondents accessed the calculator through an iframe built into the survey, so they did not leave the secure site. Once the 10-year CVD risk was calculated, participants were instructed to enter the risk value from the calculator as a response in the survey. The survey yielded a data file with no identifying information.

The risk value participants entered was used to generate 2 icon arrays, one presenting their 10-year risk of a CVD event without statin therapy and the other their risk with statin therapy. Icon arrays were displayed using a denominator of 100. The absolute risk with statin therapy was estimated from a systematic review and meta-analysis that was conducted supporting the US Preventive Services Task Force 2016 recommendation statement.^7^ The point estimate of the relative risk for CVD events from that analysis was 0.72 (95% CI, 0.63-0.81). We used a 25% risk reduction to simplify calculations and icon array presentations (rounding up to a higher risk when necessary), a figure well within the reported CI. This risk reduction estimate is also intermediate between the effect of lower-dose and higher-dose statin therapy in the Cholesterol Treatment Trialists’ Collaboration individual patient-level meta-analysis.^8,9^

Participants were then presented the possible adverse effects of statins based on meta-analyses of trials in which participants used a statin or placebo for approximately 5 years.^9^ These risks included severe muscle injury, development of diabetes, hemorrhagic stroke, and other possible adverse effects (eAppendix in the Supplement). All adverse effects were displayed using a denominator of 1000. The presentation of risks was conservative in that only risks shown to occur more often with statins than placebo in randomized clinical trials were included.

After viewing the information about the benefits and harms of the medications, participants were asked about their preference for choosing statin therapy (definitely take a statin, probably take a statin, probably not take a statin, and definitely not take a statin). Participants were also asked if they had ever talked with a health care professional about statin treatment (yes or no) and, if so, what the clinician recommended (take a statin, not take a statin, and no recommendation).

To evaluate how informed participants were about statin therapy, they answered 4 knowledge questions (eTable 1 in the Supplement) considered by us (a primary care physician [M.J.B.], an epidemiologist [S.B.], a survey researcher [F.J.F.], and 2 decision scientists [K.D.V. and K.S.]) to represent the key facts needed to make an informed decision about taking a statin. Three of the questions reflected content presented in the survey. A fourth question asked the participant about their personalized 10-year CVD risk without taking a statin. The correct response was based on their personalized risk generated from the calculator, and response options were categorized as 0% to 4%, 5% to 9%, 10% to 15%, and greater than 15%; any response in the range including the previously entered estimate from the risk calculator was considered correct.

Participants completed a 1-item literacy screener that asked how often someone helped them read instructions, pamphlets, or other written material from the physician (referred to as *doctor* in the survey) or pharmacy; the response options included never, rarely, sometimes, often, or always.^10^ Participants also completed the 8-item Subjective Numeracy Scale to obtain a measure of their perceived ability to perform mathematical tasks and comfort using numerical information. The scale has been reported to be valid and reliable.^11^ Higher scores indicate greater perceived numeracy.

To explore whether participants’ preferences for statin therapy differed by their level of education, literacy, perceived ability to interpret numerical information, or knowledge about statins, we examined the proportion of participants who had a risk value greater than 10% who would definitely or probably want statin therapy stratified by these variables. A value greater than 10% was chosen as the cut point of interest because these individuals would fall into a higher-risk category based on published guidelines.^3,4^

### Statistical Analysis

Descriptive statistics were used to characterize the study sample and their responses to survey items. Desire to use statin therapy was recoded; those who stated they definitely or probably wanted to take a statin were considered as wanting a statin, and those who stated that they definitely or probably did not want to take a statin were considered as not wanting a statin. The proportion of respondents who wanted to take a statin was calculated and is presented with 95% CIs. The Cochran-Armitage χ^2^ test for trend was used to determine whether the proportion of participants wanting to take a statin increased or decreased across ordered categories of participant characteristics. Analyses regarding statin preferences and previous statin discussion with health care professionals used χ^2^ tests, with the subgroup with less than or equal to 5% risk requiring a Fisher exact test owing to small sample sizes. Missing data were excluded pairwise. All analyses were unpaired and used 2-sided tests, with a significance threshold of *P* < .05. Statistical calculations were performed in R, version 3.5.2 (R Foundation for Statistical Computing).^12^

## Results

Data were collected on 309 participants who completed the survey, but 3 respondents who did not answer the question about their statin preference were excluded, leaving 306. Given how the opt-in panel was derived, a response rate could not be calculated. Of panel members who initiated the survey, 10 106 were not eligible, 836 were screened out because the quotas to ensure demographic diversity were already filled, and 11 were excluded because they finished the survey in less than 6 minutes. The survey was completed in a median of 12.3 minutes (interquartile range, 9.0-17.0 minutes). The 2 longest values were considered outliers based on *z* scores greater than 3 and were excluded, leaving 304 in the final sample. Eighty-six percent of the items had less than 3% missing data, including items participants chose not to answer. The knowledge items had the most missing data, varying from 6% to 8% by question. Of the 304 participants in the final sample, the cohorts were distributed equally by sex (152 [50%]) and ranged in age from 40 to 74 years, with a mean (SD) of 54.8 (9.9) years. A total of 130 participants (42.8%) were non-White, 50 (16.6%) had a high school degree or less education, and 153 (50.8%) reported never needing help reading health materials; other characteristics appear in Table 1.

**Table 1.** Demographic Characteristics of the 304 Participants in the Final Sample

Characteristic	No. (%)
Age, mean (SD), y	54.8 (9.9)
Sex	
Women	152 (50.0)
Men	152 (50.0)
Race/ethnicity^a^	
White	174 (57.2)
African American	34 (11.2)
Asian	71 (23.4)
Native Hawaiian or Pacific Islander	1 (0.3)
Other or multiple races^b^	11 (3.6)
Hispanic	21 (6.9)
Education (n = 302)	
High school/GED or less	50 (16.6)
Some college	103 (34.1)
College graduate	82 (27.2)
>4-y College degree	67 (22.2)
How often does someone help you read instructions, pamphlets, or other written material from your doctor or pharmacy? (n = 301)	
Never	153 (50.8)
Rarely	58 (19.3)
Sometimes	41 (13.6)
Often	32 (10.6)
Always	17 (5.6)

Abbreviation: GED, general educational development.

^a^ Does not total 100% because participants could choose more than 1 category.

^b^ Other includes not specified (n = 5) and multiple races (n = 6).

The mean 10-year CVD risk, as well as median and interquartile range, are presented in Table 2. When asked their preference for statin therapy after reviewing the benefit and risk information, 137 participants (45.1%) reported they would definitely (26 [8.6%]) or probably (111 [36.5%]) want to take a statin. The Figure presents the proportion of participants who would choose statin therapy across the spectrum of risk. As the risk increased, the proportion who would choose statin therapy generally increased (from 31.1% for a risk <5% to 82.6% for a risk >50%); however, the 95% CIs around the point estimates were relatively broad.

**Table 2.** Risk Value, Preference, and Patient Characteristics Associated With Interpreting Medical Information

Variable	No. (%)
10-y CVD risk value	
Mean (SD)	19.5 (26.2)
Median (IQR)	7.4 (2.4-23.2)
Preferences for or against statin therapy	
Definitely take	26 (8.6)
Probably take	111 (36.5)
Probably not take	91 (29.9)
Definitely not take	76 (25.0)
Participants reporting a health care provider ever talked about statin treatment	80 (26.3)
Of the 80 patients who ever discussed statins with their physician, these were the recommendations	
Health care professional recommended taking a statin	60 (75.0)
Health care professional recommended not taking a statin	12 (15.0)
Health care professional did not make a recommendation	8 (10.0)
Subjective Numeracy Scale, mean (SD)^a^	38.8 (6.6)
Knowledge score, mean (SD)^b^	55.8 (32.4)
Knowledge score, % correct	
0	38 (12.7)
25	54 (18.0)
50	65 (21.7)
75	87 (29.0)
100	56 (18.7)

Abbreviations: CVD, cardiovascular disease; IQR, interquartile range.

^a^ Subjective Numeracy Scale: possible score range, 8 to 48; higher score indicates higher perceived ability to perform various mathematical tasks and preference for the use of numerical vs prose information.

^b^ Four participants missed 3 or 4 of the knowledge items and so were not scored; total n = 300.

**Figure.**
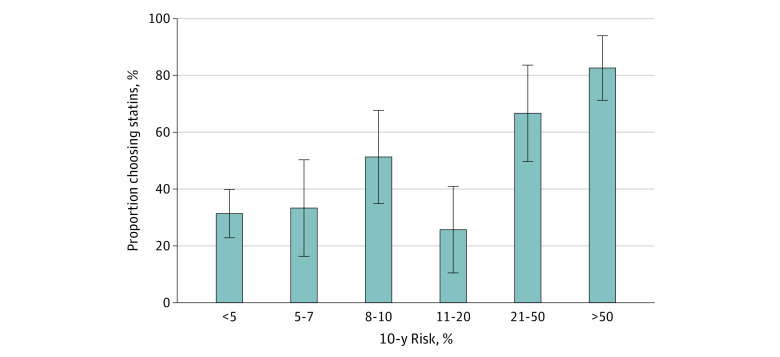
Proportion of Participants Wanting Statin Therapy, by 10-Year Estimated Risk of Cardiovascular Disease Error bars indicate 95% CIs.

When asked if a health care provider (as phrased in the survey) had ever talked with the participant about statin treatment, 80 participants (26.3%) responded yes. Of those, 60 (75.0%) responded that the recommendation was to take a statin. Total knowledge scores ranged from 0 to 100, with a mean (SD) of 55.8 (32.4) (eTable 1 in the Supplement provides details).

Table 3 presents the percentage of respondents who would choose statin therapy if minimum 10-year CVD risk thresholds were set at various levels, starting at 5%. The preferences for statins increased across increasing minimum risk thresholds (≥5%, ≥7.5%, ≥10%, ≥15%, ≥20%, and ≥25%). As the minimum risk threshold increased, more participants wanted statin therapy. Only 54.7% (95% CI, 47.4%-61.9%) of respondents with a CVD risk of 5% or higher would want statin therapy. The minimum risk threshold had to increase to 20% before 75% of respondents in that risk group would want statin therapy.

**Table 3.** Proportion of Participants Who Definitely or Probably Would Choose Statin Therapy

10-y CVD risk	Participants who would definitely/probably choose statin therapy, No.	Proportion (95% CI)
All respondents	137/304	45.1 (39.5-50.7)
Risk <5%	38/123	30.9 (22.7-39.1)
Risk thresholds		
≥5%	99/181	54.7 (47.4-61.9)
≥7.5%	85/146	58.2 (50.2-66.2)
≥10%	71/120	59.2 (50.4-68.0)
≥15%	62/93	66.7 (57.1-76.2)
≥20%	60/80	75.0 (65.5-84.5)
≥25%	60/74	81.1 (72.2-90.0)

Abbreviation: CVD, cardiovascular disease.

Statin preferences depended to some degree on whether the participant reported a previous statin discussion with a health care professional (eTable 2 in the Supplement). As the risk threshold increased, the proportion of participants who reported a discussion about statin therapy increased: greater than 5%, 67 of 181 (37.0%); greater than 7.5%, 61 of 146 (41.8%); greater than 10%, 51 of 120 (42.5%); greater than 15%, 48 of 93 (51.6%); greater than 20%, 45 of 80 (56.2%); and greater than 25%, 43 of 74 (58.1%). In the overall sample and in the subset of participants with a 10-year CVD risk greater than 5%, a previous discussion was associated with a significantly higher proportion of participants wanting a statin (overall sample: χ^2^_1_ = 20.86, *P* < .001; >5%: χ^2^_1_ = 9.29, *P* = .002).

Table 4 reports on associations between wanting statin therapy and education, literacy, numeracy, and knowledge for patients whose risk was greater than 10%. Education and wanting statin therapy were not significantly associated (χ^2^_4_ = −0.55, *P* = .58). However, there was a significant trend indicating that, as participants’ health literacy increased, they were less likely to want statin therapy (χ^2^_4_ = 2.91, *P* = .004). Similarly, as subjective numeracy and knowledge scores increased, participants were less likely to want statin therapy (subjective numeracy: χ^2^_4_ = 2.58, *P* = .01; knowledge scores: χ^2^_5_ = 3.52, *P* < .001).

**Table 4.** Proportion of Participants With a Risk Value Greater Than 10% Who Definitely or Probably Would Choose Statin Therapy

Variable	Definitely/probably want statin therapy, No. (%)	*P* value for χ^2^ test for trend
Education		
High school/GED or less	16/27 (59.3)	.58
Some college	24/47 (51.1)	
4-y College degree	21/26 (80.8)	
>4-y College degree	10/19 (52.6)	
1-Item literacy screener		
Always/often	16/25 (64.0)	.004
Sometimes	24/29 (82.8)	
Rarely	13/20 (65.0)	
Never	17/44 (38.6)	
Subjective Numeracy Scale		
1st Quartile (lowest)	21/30 (70.0)	.01
2nd Quartile	18/30 (60.0)	
3rd Quartile	24/32 (75.0)	
4th Quartile (highest)	8/28 (28.6)	
Knowledge questions answered correctly, No. (%)		
0	27/32 (84.4)	<.001
25	19/28 (67.9)	
50	10/23 (43.5)	
75	7/23 (30.4)	
100	8/14 (57.1)	

Abbreviation: GED, general educational development.

## Discussion

In this study, we examined whether participants would want to use statin therapy for primary prevention of CVD after reviewing their 10-year cardiovascular risk, their estimated absolute risk reduction, and the adverse effects associated with statin therapy. We found that the proportion of participants who definitely or probably would choose statin therapy after reviewing their personalized information was generally flatter than expected across risk thresholds, particularly from greater than or equal to 5% (54.7%) to greater than or equal to 10% (59.2%) (Table 3 and Figure). At the lower end of the thresholds, 30.9% of participants with a risk below 5% wanted statin therapy, and at the higher end, only 66.7% of participants with a risk greater than or equal to 15% wanted statin therapy. We could not find a risk threshold that would justify a standard for or against statin therapy according to the Eddy^1^ definition requiring at least 95% of patients wanting or not wanting statin therapy. These data suggest that the range of risk values for which shared decision-making is appropriate may be much broader than current guidelines suggest.

We wondered whether better educated, more health literate, more numerate, and more knowledgeable participants with a 10-year CVD risk greater than 10%, which is the level at which guidelines generally recommend statin therapy, might be more enthusiastic about statin therapy. We found the opposite in this sample. Educational level was unrelated to wanting statin therapy, and more health literate, more numerate, and more knowledgeable participants in this risk group were less likely to want statin therapy (Table 4). These data suggest that providing patients with more information to help them make better decisions may decrease their enthusiasm for use of statin therapy.

Some early guidelines, including a guideline on management of benign prostatic hyperplasia developed by the American Urological Association, used the Eddy approach.^13^ As part of guideline development, a balance sheet addressing surgery, medication, and watchful waiting was presented to men in the practices of panel members. This analysis showed that few men with a low symptom score wanted treatment; therefore, a standard for watchful waiting for these patients was recommended. Active treatment was considered optional because no preponderance of patients wanted treatment, even with high symptom scores.

In addition to Eddy, Montori and colleagues^14^ have called for the incorporation of patient preferences into guidelines, although without proposing how to do so. The Grading of Recommendation, Assessment, Development and Evaluation group approach to eliciting values and preferences focuses on the importance people place on health outcomes rather than their preferences for particular treatments.^15^ Yebyo and colleagues^16^ surveyed 220 people in Ethiopia and Switzerland about the relative desirability of statin benefits and harms; however, this study did not directly elicit preferences for statin treatment. Another approach used for incorporating patient preferences into guidelines is deliberative democracy, such as citizen juries, in which groups of people learn about the benefits and harms of interventions and express their informed preferences.^17,18^ However, these methods are labor intensive and often involve relatively few people in the deliberation. If the preference distributions we generated with nonprobability internet panels can be replicated in a clinical population, our method of directly eliciting preference distributions for treatments over a spectrum of risk may be a relatively efficient means of collecting preference data for use in guideline development.

### Limitations

This study has several limitations. The number of participants in different 10-year CVD risk strata was low, resulting in relatively broad 95% CIs around point estimates of the proportion of participants wanting statin therapy (Figure). Participants supplied their cholesterol and blood pressure values, and we do not know whether their entries were accurate. Our participants had not recently received a statin, and they may represent a population less likely to be interested in statin therapy. However, only 26.3% of participants reported a previous discussion with a clinician about statin therapy, and among those with previous discussion, interest in statin therapy was generally low, especially at lower risk thresholds (eTable 2 in the Supplement). The adverse effects denominator was 1000 and the effectiveness denominator was 100, which could have resulted in bias toward overemphasizing the risks. In addition, although our sample of participants was relatively diverse (Table 1), we do not know whether this internet sample of survey volunteers was representative of the broader patient population who might consider statin therapy. White and Latinx people were underrepresented and Asian people were overrepresented in our sample compared with US Census figures.^19^

In addition, the preference distribution of participants may have been influenced by our conservative presentation of statin adverse effects, reflecting only the risks seen more commonly than placebo in clinical trials (eAppendix in the Supplement). If we had instead presented the probabilities of potential adverse effects in observational studies, in which as many as 11% to 29% of patients reported muscle symptoms,^20^ the enthusiasm for statin therapy might have been even lower.

## Conclusions

In this survey study, participants’ preferences for statin therapy for primary prevention of CVD who are not currently receiving statin or proprotein convertase subtilisin/kexin type 9 inhibitor therapy varied across the spectrum of estimated 10-year cardiovascular risk. These findings may have implications for clinical practice guidelines, and they suggest a relatively broad range of CVD risk, perhaps 5% to 15% or higher, over which to recommend shared decision-making between clinicians and patients about statin therapy. Given these findings, it will be important to replicate this study with larger numbers, including clinical populations. If these findings are confirmed, they could have important implications for clinical guidelines and practice.

## Notes

Supplement. **eTable 1.** Knowledge Questions and Responses **eTable 2.** Proportion of Participants Who Definitely or Probably Wanted to Take a Statin Based on Risk Thresholds and Whether or Not They Reported a Previous Discussion With a Health Care Provider About Taking a Statin **eAppendix.** Statin Preference Survey
