# Supplementary material for: Patient Preference Distribution for Use of Statin Therapy
Source: JAMA Netw Open. 2021 Mar 15;4(3):e210661. doi: 10.1001/jamanetworkopen.2021.0661 (PMC7961307; doi:10.1001/jamanetworkopen.2021.0661)
Supplement: Supplement. — eTable 1. Knowledge Questions and Responses eTable 2. Proportion of Participants Who Definitely or Probably Wanted to Take a Statin Based on Risk Thresholds and Whether or Not They Reported a Previous Discussion With a Health Care Provider About Taking a Statin eAppendix. Statin Preference Survey [file jamanetwopen-e210661-s001.pdf]

Supplementary Online Content

Brodney S, Valentine KD, Sepucha K, Fowler FJ Jr, Barry MJ. Patient preference distribution for use of statin therapy. *JAMA Netw Open*. 2021;4(3):e210661. doi:10.1001/jamanetworkopen.2021.0661

eTable 1. Knowledge Questions and Responses

eTable 2. Proportion of Participants Who Definitely or Probably Wanted to Take a Statin Based on Risk Thresholds and Whether or Not They Reported a Previous Discussion With a Health Care Provider About Taking a Statin

eAppendix. Statin Preference Survey

This supplementary material has been provided by the authors to give readers additional information about their work.

© 2021 Brodney, S, et al. *JAMA Network Open.*

eTable 1. Knowledge Questions and Responses

Knowledge Questions and Responses	% selecting each option
Question 1. The benefit of taking a statin is greater if:	
○ I've taken a statin before	12%
○ My risk of having a heart attack or stroke is higher to begin with (correct)	54%
○ My risk of having a heart attack or stroke is lower to begin with	21%
○ I have muscle aches on the statin	6%
○ I do not want to answer this question.	7%
**Question 2. Based on my risk factors, my 10-year risk of having a heart attack or stroke WITHOUT taking a statin is:**	**N correct/N choosing the answer (%)***
○ 0-4%	98/109 (90%)
○ 5-9%	52/96 (54%)
○ 10-15%	26/60 (43%)
○ Greater than 15%	29/38 (76%)
○ I do not want to answer this question.	0/1 (0%)
**Question 3. Which of the following is a side effect of taking a statin?**	**% selecting each option**
○ Heart failure	8%
○ Kidney disease	13%
○ Migraines	14%
○ Diabetes (correct)	57%
○ I do not want to answer this question.	8%
**Question 4. Statins can cause serious muscle problems, such as pain, tenderness or weakness. About how many people who take a statin develop these serious muscle problems?**	**% selecting each option**
○ About 1 in 1000 (correct)	42%
○ About 10 in 1000	32%
○ About 100 in 1000	13%
○ About 500 in 1000	7%
○ I do not want to answer this question.	5%

*Response was based on individual's risk. Overall, 68% were able to correctly recall their risk category.

© 2021 Brodney, S, et al. *JAMA Network Open.*

eTable 2. Proportion of participants who definitely or probably wanted to take a statin based on risk thresholds and whether or not they reported a previous discussion with a health care provider about taking a statin.

Risk	N discussed with clinician	Proportion discussed with clinician (95% CI)	Discussion, N wanting statin	Discussion, Proportion wanting statin (95% CI)	No Discussion, N wanting statin	No Discussion, Proportion wanting statin (95% CI)	Unadjusted p-value*
All Patients	80/304	26.3% (21.4-31.3)	54/80	67.5% (57.2-77.8)	83/224	37.1% (30.7-43.4)	0.0
Risk >5%	67/181	37.0% (30-44.1)	47/67	70.1% (59.2-81.1)	52/114	45.6% (36.5-54.8)	0.002
Risk >7.5%	61/146	41.8% (33.8-49.8)	43/61	70.5% (59-81.9)	42/85	49.4% (38.8-60)	0.017
Risk >10%	51/120	42.5% (33.7-51.3)	37/51	72.5% (60.3-84.8)	34/69	49.3% (37.5-61.1)	0.017
Risk >15%	48/93	51.6% (41.5-61.8)	35/48	72.9% (60.3-85.5)	27/45	60% (45.7-74.3)	0.271
Risk >20%	45/80	56.2% (45.4-67.1)	35/45	77.8% (65.6-89.9)	25/35	71.4% (56.5-86.4)	0.696
Risk >25%	43/74	58.1% (46.9-69.3)	35/43	81.4% (69.8-93)	25/31	80.6% (66.7-94.6)	1

*As the rows of this table are not mutually exclusive, and given the multiple comparisons, we used a Bonferroni Correction to adjust the level of significance required to conclude a finding was unlikely to be due to chance to P=0.05/7=0.007.

© 2021 Brodney, S, et al. *JAMA Network Open.*

eAppendix. Statin Preference Survey

Welcome to the Statin Preference Survey! The purpose of the survey is to explore how different people might think about whether or not to use a statin medicine to lower the risk of a heart attack or stroke. This research project is being conducted by researchers at Massachusetts General Hospital. The following screen contains information about the study. We hope you take the time to participate.

What is the purpose of the research study? The purpose of the study is to explore how different people think about whether or not to use a statin medicine to lower the risk of a heart attack or stroke. The amount of benefit a person will get from taking a statin will depend on their risk of a heart attack or stroke to begin with. People with a high risk will get more benefit than those at low risk.

What is involved in the research study? Participants will be asked to complete a survey. It should take less than 10 minutes to complete. Your participation is completely voluntary (your choice). You may decide not to participate. You may skip any question for any reason. No confidential or protected health information (PHI) will be collected as part of this study.

What are the risks or discomforts of the research study? There are risks to taking part in any research study. There are very small risks to subjects in this research study. The main risk is that you may feel uncomfortable answering some of the questions in the survey. If you do not wish to answer any question, for any reason, you do not have to. The research team will make every effort to keep all the information you share strictly confidential, as required by law. The researchers will never have access to any identifying information. Your answers will be combined with answers from other respondents and the information will not be presented or published in any way that would allow you to be identified.

What are the benefits of the research study? Participants should not expect any benefits from completing the survey. However, some people may see their participation as beneficial because it will help create a survey to be used in future research projects aimed at improving the quality of medical decisions.

Whom do I contact if I have questions about the research study? If you have questions about the research study, please contact:

Suzanne Brodney at 617-643-4266 or sbrodney@mgh.harvard.edu

Dr. Michael Barry, Principal Investigator, at 617-726-5211 or mbarry@mgh.harvard.edu

If you'd like to speak to someone not involved in this research about your rights as research subject, or any concerns or complaints you may have about the research, contact the Partners Human Research Committee at 857-282-1900.

© 2021 Brodney, S, et al. *JAMA Network Open.*

I agree to participate in this research.

○ Yes

○ No

To begin, we would like to give you some information about high cholesterol and a medicine called a statin. A statin is a type of medicine used to lower high cholesterol. Lowering high cholesterol levels can lower the risk of having a heart attack or stroke. Heart attacks and strokes are caused by blocked blood vessels to the heart or brain. We want to understand how different people think about whether or not to use a statin medicine to lower the risk of a heart attack or stroke. The amount of benefit a person will get from taking a statin will depend on their risk of a heart attack or stroke to begin with. People with a high risk will get more benefit than those at low risk.

A person deciding about a statin needs to weigh the benefit against the side effects of taking a statin pill every day. This survey has several parts.

First, we will have you enter some information to calculate your risk of having a heart attack or stroke.

Second, we will give you some information about benefits of taking a statin and some side effects and harms of taking a statin.

Third, we will ask you some questions about the information and your opinion about taking a statin. Click the arrow to begin.

What was your age on your last birthday?

____

© 2021 Brodney, S, et al. *JAMA Network Open.*

Which best describes your gender identity?

- ◯ Male
- ◯ Female
- ◯ Something else ________

What is your race/ethnic background?

- ◯ White, not Hispanic
- ◯ Black, not Hispanic
- ◯ Hispanic
- ◯ Something else

What is the highest grade or level of school that you have completed?

- ◯ 8th grade or less
- ◯ Some high school, but did not graduate
- ◯ High school graduate or GED
- ◯ Some college or 2-year degree
- ◯ 4-year college graduate
- ◯ More than a 4-year college degree

© 2021 Brodney, S, et al. *JAMA Network Open.*

Do you know what your total cholesterol number is?

- ◯ Yes
- ◯ No

Did you have your total cholesterol tested in the last 3 years?

- ◯ Yes
- ◯ No

Do you know what your HDL (the "good") cholesterol number is?

- ◯ Yes
- ◯ No

Do you know what your blood pressure is?

- ◯ Yes
- ◯ No

Did you have your blood pressure tested in the last 3 years?

- ◯ Yes
- ◯ No

© 2021 Brodney, S, et al. *JAMA Network Open.*

We would like to know if you are currently taking a statin medication or if you have you been on a statin medication within the last 3 years. Below is a list of statin medications.

Statin Medications:

atorvastatin (Lipitor)

fluvastatin (Lescol)

lovastatin (Mevacor, Altocor)

pravastatin (Pravachol)

pitavastatin (Livalo)

simvastatin (Zocor)

rosuvastatin (Crestor)

Have you taken any of those in the past 3 years?

- ◯ Yes
- ◯ No

We would like to know if you are currently taking a PCSK9 inhibitor or have you been on a PCSK9 inhibitor within the last 3 years. Below is a list of PCSK9 inhibitors. These are injections that you get in a medical office or give yourself.

PCSK9 inhibitors:

Evolocumab (Repatha)
Alirocumab (Pradulent)

Have you taken either of those in the past 3 years?

- ◯ Yes
- ◯ No

© 2021 Brodney, S, et al. *JAMA Network Open.*

As far as you know, are you allergic to a statin medication?

○ Yes

○ No

Have you ever had: (Mark all that apply.)

☐ A heart attack

☐ A stroke

☐ Blockages in a blood vessel in your neck or head

☐ A coronary artery bypass operation (CABG)

☐ A stent placed in one or more of your arteries

☐ None of these

This next set of questions will ask you to enter some information in a risk calculator. You will need to enter your total cholesterol, HDL ("good") cholesterol and systolic blood pressure. Please answer each question. Once you enter your information, please click the CALCULATE button. This will give you your 10-year risk of atherosclerotic cardiovascular disease. Please enter this number in the space provided at the bottom of the page.

Total cholesterol: Most people have total cholesterol numbers in the range of 130 to 320. Please enter your number. If your number is less than 130, please enter 130. If your number is higher than 320, please enter 320.

HDL cholesterol: Most people have HDL cholesterol numbers in the range of 20 to 100. Please enter your number. If your number is less than 20, please enter 20. If your number is higher

© 2021 Brodney, S, et al. *JAMA Network Open.*

than 100, please enter 100.

<u>Systolic blood pressure:</u> Blood pressure is reported as two numbers. We are interested in the first number, which is the higher number. Most people have systolic blood pressure numbers in the range of 90 to 200. Please enter your number. If your number is less than 90, please enter 90. If your number is higher than 200, please enter 200.

When you put your information in the calculator above you get both your 10-year risk of atherosclerotic cardiovascular disease, as well as other information. We are **ONLY** interested in your 10-year risk of atherosclerotic cardiovascular disease. To help you find the number we are looking for, we included an example of what the screen looks like below. In this example, the number we are looking for is in the red circle. It is located under the blue print button.

Please find YOUR number that appears in the calculator above and enter it in the text box below. You do not need to enter the % sign.

____

© 2021 Brodney, S, et al. *JAMA Network Open.*

Now that we've informed you about the possible benefits of taking a statin, let's tell you about the possible side effects and harms. This information about side effects and harms come from studies where people have taken a statin for about 5 years.

Severe muscle problems

Statins can cause severe muscle problems, such as pain, tenderness or weakness.

For every 1000 people treated with a statin, less than 1 person will get severe muscle problems. That means more than 999 out of 1000 people won't get severe muscle problems. Once the statin is stopped, the problem usually goes away quickly.

Diabetes

Statins can increase the risk of developing diabetes. For every 1,000 people treated with a statin, between 5 and 10 people will develop diabetes. That means 990 to 995 people won't get diabetes.

Stroke

Statins prevent the most common type of stroke. However, taking a statin may increase the risk of a less common type of stroke, which happens when a blood vessel in the brain leaks. For every 1,000 people who take a statin, less than 1 person will have this kind of stroke. That means more than 999 people out of 1000 won't get this uncommon type of stroke.

Other possible side effects

In clinical studies, patients taking statins reported the following common side effects: diarrhea, upset stomach, mild joint or muscle pain, tiredness, tendon problems, memory loss, and confusion. When the patients taking statins were compared to patients taking a placebo pill (a fake pill that looks like a statin), both groups of patients reported side effects at about the same rates. This means it is not clear if these reported side effects were caused by the statins.

© 2021 Brodney, S, et al. *JAMA Network Open.*

The next 4 questions have some information that doctors and other patients feel is important to understand about the risks and benefits of statins. The correct answers are based on medical research. You may not know the exact answer, but please take your best guess.

The benefit of taking a statin is greater if:

- ◯ I've taken a statin before
- ◯ My risk of having a stroke or heart attack is higher to begin with
- ◯ My risk of having a heart attack or stroke is lower to begin with
- ◯ I have muscle aches on the statin
- ◯ I do not want to answer this question.

Based on my risk factors, my 10-year risk of having a heart attack or stroke WITHOUT taking a statin is:

- ◯ 0-4%
- ◯ 5-9%
- ◯ 10-15%
- ◯ Greater than 15%
- ◯ I do not want to answer this question.

© 2021 Brodney, S, et al. *JAMA Network Open.*

Which of the following is a side effect of taking a statin?

- Heart failure
- Kidney disease
- Migraines
- Diabetes
- I do not want to answer this question.

Statins can cause serious muscle problems, such as pain, tenderness or weakness. About how many people who take a statin develop these serious muscle problems?

- About 1 in 1000
- About 10 in 1000
- About 100 in 1000
- About 500 in 1000
- I do not want to answer this question.

© 2021 Brodney, S, et al. *JAMA Network Open.*

Assume your healthcare provider wants to know how interested you are in taking a statin. Based on what we've told you, what do you think you would want to do?

- ○ Definitely take a statin
- ○ Probably take a statin
- ○ Probably not take a statin
- ○ Definitely not take a statin
- ○ I do not want to answer this question.

Has a healthcare provider ever talked about statin treatment with you?

- ○ Yes
- ○ No
- ○ I do not want to answer this question.

What did your healthcare provider recommend you do about taking a statin?

- ○ Take a statin
- ○ Not take a statin
- ○ My healthcare provider did not make a recommendation.
- ○ I do not want to answer this question.

© 2021 Brodney, S, et al. *JAMA Network Open.*

How big did the benefit of taking a statin feel to you?

- ◯ Felt like a large benefit
- ◯ Felt like a medium benefit
- ◯ Felt like a small benefit
- ◯ Felt like almost no benefit
- ◯ I do not want to answer this question.

How much did the possible side effects of taking a statin worry you?

- ◯ Felt very worried
- ◯ Felt somewhat worried
- ◯ Felt a little worried
- ◯ Felt not worried at all
- ◯ I do not want to answer this question.

© 2021 Brodney, S, et al. *JAMA Network Open.*

How often does someone help you read instructions, pamphlets or other written material from your doctor or pharmacy?

- ◯ Never
- ◯ Rarely
- ◯ Sometimes
- ◯ Often
- ◯ Always
- ◯ I do not want to answer this question.

For each of the following questions, please check the box that best reflects **how good you are at doing the following things**:

How good are you at working with fractions?

- ◯ 1 - Not at all good
- ◯ 2
- ◯ 3
- ◯ 4
- ◯ 5
- ◯ 6 - Extremely good
- ◯ I do not want to answer this question.

© 2021 Brodney, S, et al. *JAMA Network Open.*

How good are you at working with percentages?

- ◯ 1 - Not at all good
- ◯ 2
- ◯ 3
- ◯ 4
- ◯ 5
- ◯ 6 - Extremely good
- ◯ I do not want to answer this question.

How good are you at calculating a 15% tip?

- ◯ 1 - Not at all good
- ◯ 2
- ◯ 3
- ◯ 4
- ◯ 5
- ◯ 6 - Extremely good
- ◯ I do not want to answer this question.

How good are you at figuring out how much a shirt will cost if it is 25% off?

- ◯ 1 - Not at all good
- ◯ 2
- ◯ 3

© 2021 Brodney, S, et al. *JAMA Network Open.*

- ○ 4
- ○ 5
- ○ 6 - Extremely good
- ○ I do not want to answer this question.

© 2021 Brodney, S, et al. *JAMA Network Open.*

For each of the following questions, please check the box that **best reflects your answer**:

When reading the newspaper, how helpful do you find tables and graphs that are parts of a story?

- ◯ 1 - Not at all helpful
- ◯ 2
- ◯ 3
- ◯ 4
- ◯ 5
- ◯ 6 - Extremely helpful
- ◯ I do not want to answer this question.

When people tell you the chance of something happening, do you prefer that they use **words** ("it rarely happens") or **numbers** ("there's a 1% chance")?

- ◯ 1 - Always prefer words
- ◯ 2
- ◯ 3
- ◯ 4
- ◯ 5
- ◯ 6 - Always prefer numbers
- ◯ I do not want to answer this question.

© 2021 Brodney, S, et al. *JAMA Network Open.*

When you hear a weather forecast, do you prefer predictions using **percentages** (e.g., "there will be a 20% chance of rain today") or predictions using only **words** (e.g., "there is a small chance of rain today")?

- ◯ 1 - Always prefer percentages
- ◯ 2
- ◯ 3
- ◯ 4
- ◯ 5
- ◯ 6 - Always prefer words
- ◯ I do not want to answer this question.

How often do you find numerical information to be useful?

- ◯ 1 - Never
- ◯ 2
- ◯ 3
- ◯ 4
- ◯ 5
- ◯ 6 - Very often
- ◯ I do not want to answer this question.

© 2021 Brodney, S, et al. *JAMA Network Open.*

Thinking about how much healthcare you prefer to get: What type are you? Sometimes, medical action is clearly necessary and sometimes it is clearly NOT necessary. Other times, reasonable people differ in their beliefs about whether medical action is needed. In situations where it's not clear, do you tend to lean toward taking action or do you lean towards waiting and seeing if action is needed Importantly, there is no "right" way to be.
Please answer on the 1-6 scale below:

- ○ 1 - I strongly lean toward waiting and seeing
- ○ 2
- ○ 3
- ○ 4
- ○ 5
- ○ 6 - I strongly lean toward taking action
- ○ I do not want to answer this question.

In addition to being Hispanic, do you consider yourself to be any other race or ethnicity?

- ○ Yes
- ○ No
- ○ I do not want to answer this question.

© 2021 Brodney, S, et al. *JAMA Network Open.*

We'd like to ask you more about your race. Which of the following do you consider yourself? Please choose as many as you feel describe you.

- ☐ White
- ☐ Black or African American
- ☐ Asian
- ☐ Native Hawaiian or Other Pacific Islander
- ☐ American Indian or Alaska Native
- ☐ Other
- ☐ I do not want to answer this question.

Please let us know any thoughts you had while taking the survey or any problems you encountered.

____

____

Thank you! You are done with the survey. We have done our best to give you an accurate presentation on the possible benefits and harms of taking a statin. This presentation depends on the information you entered at the beginning of the survey. Of course, before making any decisions about taking or not taking a statin, you should discuss your options with your own clinician.

If you want more information about estimating your risk of a heart attack or stroke, go here: https://clincalc.com/Cardiology/ASCVD/PooledCohort.aspx

If you want more information about cholesterol and statins, go here: https://www.fda.gov/media/82811/download

© 2021 Brodney, S, et al. *JAMA Network Open.*
